# Unveiling the role of dual grading in device optimization of HTL-free Sb_2_(S, Se)_3_ solar cells

**DOI:** 10.1038/s41598-025-11658-8

**Published:** 2025-07-25

**Authors:** Basma A. A. Osman, Ahmed Shaker, Ibrahim S. Ahmed, Tarek M. Abdolkader

**Affiliations:** 1 Department of Basic Engineering Sciences, Benha Faculty of Engineering, Benha University, Benha, Egypt; 2 Department of Engineering Physics and Mathematics, Faculty of Engineering, Ain Shams University, Cairo, Egypt

**Keywords:** Sb_2_(S,Se)_3_, Bandgap grading, Doping grading, CBO, CdZnS, HTL free, Energy science and technology, Materials science, Optics and photonics, Physics

## Abstract

Antimony Seleno-sulfide, Sb_2_(S, Se)_3_, is considered an extremely promising absorber for optoelectronic applications regarding its minor toxicity, phase stability, earth-abundancy besides its decent absorption capabilities. Moreover, it possesses tunable bandgaps which can be adjusted from 1.1 to 1.7 eV by varying the Se/S ratio in Sb_2_(S, Se)_3_ alloy films, making it an attractive candidate for efficient solar cells. This study proposes novel designs for hole-transporting layer (HTL)-free Sb_2_(S, Se)_3_ solar cells that enhance photo-generated carrier collection, thereby improving power conversion efficiency (PCE). Utilizing SCAPS-1D solar cell simulator, we first validate the device model by calibrating an experimental cell with the FTO/CdS/Sb_2_(S, Se)_3_/Au configuration. The primary objective is to optimize HTL-free Sb_2_(S, Se)_3_ solar cells using dual grading techniques in the electron-transport layer (ETL) and within the absorber. Our analysis reveals that the band offsets at both the ETL/absorber and ETL/front contact interfaces play competing roles in determining cell performance. To optimize these offsets, we replace the traditional CdS ETL with the tunable Cd₁₋ₓZₓS and gradually adjust the Zn fraction at both interfaces. We also conduct a comparative study of various bandgap grading (BGG) profiles (linear, logarithmic, and parabolic). A doping grading technique is also applied to the absorber to enhance charge carrier extraction. The simulation results indicate that dual grading techniques in the ETL and absorber, combined with optimized absorber thickness, can significantly improve device performance, yielding a simulated PCE of 21.15%. These findings provide valuable insights into the design and optimization of HTL-free Sb_2_(S, Se)_3_ solar cells.

**Supplementary Information:**

The online version contains supplementary material available at 10.1038/s41598-025-11658-8.

## Introduction

In recent years, the transition to renewable energy has significantly enhanced productivity and quality of life, serving as a cornerstone for economic and social development^1^. One of the most prominent types of renewable energy sources that is currently advancing quickly to keep up with the growing global energy issues is solar energy^2^. To improve power conversion efficiency (PCE), several solar cell (SC) technologies have been studied^3^. Despite advancements in technologies such as microstructures and nanowires aimed at enhancing PCE and reducing costs^4^, the efficiencies achieved with these approaches still fall short compared to those of crystalline silicon (c-Si) solar cells, known for their outstanding PCE and stability in various applications. However, thin-film solar cells are becoming a perfect substitute for Si-based solar cells thanks to their affordable prices, high efficiency, and enhanced device flexibility^5,6^. CdTe and CuInGaSe_2_ are distinguished examples of photovoltaic (PV) thin films, which are commonly utilized in flexible devices and for constructing integrated PV systems^7^. Nevertheless, their use is somewhat limited by the rarity of In, Te, and Ga as well as the toxicity of Cd. Therefore, research is necessary to find nontoxic, Earth-abundant PV materials such as Sb_2_S_3_^8^, Sb_2_Se_3_^9^, Sb_2_(S, Se)_3_^9^, CuSbSe_2_^10^, CuSbS_2_^11^, and other candidates.

The family of Antimony Chalcogenide binary compounds Sb_2_Se_3_, Sb_2_S_3_ and Sb_2_(S, Se)_3_ thin film cells has witnessed substantial progress in the last few years. An inorganic Antimony Selenide (V-VI binary compound), Sb_2_Se_3_, is a very suitable SC light absorber due to its attractive optoelectronic properties such as superior electrical conductivity, absorption coefficients larger than 10^5^ cm^−1^, nearly 60 ns carrier lifetime, and bandgap (1.03–1.17 eV)^12^ which is proper and close to the optimum value of Shockey-Queisser, aside from its rare toxicity, and appropriate price^13^. Advantages also include its single orthorhombic structure and 1D crystalline structure^14^. Additionally, antimony sulfide, Sb_2_S_3_, has impressive properties for solar cells. It is one of the most promising light-absorbers for the fact that Sb_2_S_3_ is low-cost, environmentally friendly, and abundant on earth. Besides, it has an acceptable bandgap of nearly 1.7 eV, a superior absorption coefficient of approximately 2 × 10^5^ cm^−1^, and sufficient air-stability^8^. The same crystal structure of Sb_2_S_3_ and Sb_2_Se_3_ permits tuning the bandgap of Sb_2_(S, Se)_3_ from about 1.1 eV to 1.7 eV by modifying the Se/S atomic ratio^15^. Adjusting the Se/S atomic ratio results in a more acceptable absorber bandgap for light harvesting, in addition to a compact morphology, larger grains and favorable crystal orientation^16^. The p-type Sb_2_(S, Se)_3_ semiconductor features a tunable absorption cutoff edge (730–1050 nm)^17^. Recently, the PCE of Sb_2_(S, Se)_3_-based thin films SCs reached above 10% efficiency^16^.

Choosing a suitable ETL for Sb_2_(S, Se)_3_-based SCs is extremely important to dismiss absorption losses and improve interface performance between absorber and ETL. The stability and large electron mobility of CdS make it an ideal ETL material for Sb_2_(S, Se)_3_ SCs^9^. Conversely, it confronts two serious issues: (1) absorption at long wavelengths owing to the relatively small bandgap (2.42 eV)^8,18^ and (2) the undesired recombination at the interface between CdS/Sb_2_(S, Se)_3_, due to the unoptimized band alignment between them. As a result, it was found that lowering the thickness or substituting the CdS layer with a larger gap layer such as SnO_2_, TiO_2_, ZnO, or ZnMgO are possible solutions^19^. In addition, the fabrication of double buffer layers is another possible solution; however, it seems to be extremely challenging. One suitable strategy for increasing the CdS bandgap to permit absorbing more photons in the absorber layer is to add more dopant atoms or replace Cd or S with alternative atoms. Ternary Cd_1−x_Zn_x_S (0 ≤ x ≤ 1) compound is an acceptable alternative to CdS as an ETL as it modifies electron affinity and energy bandgap^18^. The bandgap may be modified by adjusting the Zn concentration and the band alignment between buffer/absorber^18^. The role of Cd_1−x_Zn_x_S as an ETL in fabricated cells increased efficiency from 5.08 to 6.71%, as reported previously^20^. In a simulation study, the authors simulated an Au/Sb_2_(S, Se)_3_/CdS/ITO structure. The optimized cell achieved a simulated 14.86% PCE by employing Cd_0.44_Zn_0.56_S as an ETL to tune the conduction band offset (CBO) between the absorber and the ETL, as well as various other factors^18^.

Numerous methodologies, both experimental and simulation based, were applied to boost the PCE of Sb_2_(S, Se)_3_-based SCs. Kanghua Li et el., investigated a new approach to optimize the ITO/CdS/Sb_2_(S, Se)_3_/Au cell arrangement. They used vapor transport deposition (VTD) technology to design an Sb_2_(S, Se)_3_ cell with V-shaped bandgap energy. They obtained a PCE of 7.27% after optimization^17^. In 2020, a PCE of 10.0% was reported by studying a novel Se/(S + Se) atomic ratio of 29% in FTO/CdS/Sb_2_(S, Se)_3_ SCs to get an acceptable bandgap of Sb_2_(S, Se)_3_^16^. In 2021, researchers introduced a theoretical triple-junction Sb_2_S_3_/Sb_2_(_0.7_Se_0.3_)_3_/Sb_2_Se_3_ SC utilizing several band alignment strategies. Raising the concentration of Se in the Sb_2_(S_1−x_Se_x_)_3_-based solar cell resulted in a theoretical efficiency of 33%^21^. Moreover, it was discovered that an Sb_2_(S, Se)_3_ SC with a Sb_2_S_3_ mass ratio of 0.25% between Sb_2_S_3_ and the overall powder had the best crystallinity. This concept has been used in ITO/CdS/Sb_2_(S, Se)_3_/Au SCs, yielding 7.31% PCE^17^. Furthermore, Junwei Chen et al. found that applying TA-Sb_2_(S, Se)_3_ in a planar heterojunction SC yielded a PCE of 9.28% and a *V*_*oc*_ of 0.7 V^22^. To manage the chemical interaction of Sb material with Se and S in Sb_2_(S, Se)_3_, the hydrothermal temperature must be adjusted. This process resulted in a device possessing the highest PCE of 10.55% among Sb_2_(S, Se)_3_-based SCs until now^23^. In 2024, Lei Zhang et al. used alkali halide (CsI) as a precursor in hydrothermal processes to enhance the performance of antimony selenosulfide SCs. This causes a rise in PCE to 10.05%^24^. In 2023, Yue Deng et al. developed a two-step hydrothermal deposition process for making Sb_2_S_3_/Sb_2_(S, Se)_3_ films with big grains and smooth surfaces. Applying these films in SnO_2_/CdS/Sb_2_S_3_/Sb_2_(S, Se)_3_/carbon SCs yielded a PCE of 2.76%. After that, they used P_3_HT as HTL and got a PCE of 4.15%^25^. Wangchao Chen et al. constructed two HTLs (free dopant materials) named F-BDT and T-BDT. They realized that the cell F-BDT/Sb_2_(S, Se)_3_ achieved 9.13% efficiency^26^.

Although SCs utilizing organic HTL materials, such as P_3_HT_5_ and Spiro-OMeTAD, achieved a high PCE^27^, these HTL materials have undesirable features, such as low long-term stability and high fabrication cost^27^. To deal with these issues, HTL-free SCs have been introduced to the PV community^28^. These cells would lower interface defects between HTL and absorber as well as fabrication expenses^2,27^. For instance, HTL-free Sb_2_(S, Se)_3_ SC structure was manufactured by VTD process with E_g_ = 1.33 eV, achieving a PCE of 7.03%^28^. To further increase PCE in the free HTL design, the CBO between the ETL and the absorber needs to be aligned. This alignment may be achieved by employing an adjustable bandgap ETL material such as Cd_1−x_Zn_x_S^29^.

One of the most critical factors in an n-i-p heterojunction is the precise band alignment, not only between the absorber layer and the ETL, but also between the ETL and the front contact. Proper band alignment at both interfaces is essential to minimize energy losses, enhance carrier extraction, and prevent recombination. Through the adjustment of bands, a smoother transportation of electrons from the absorber through the ETL to the front contact (FC) can be obtained, resulting in boosting the PCE of the SC. Thus, in this paper, we introduce a design of a single junction HTL-free Sb_2_(S, Se)_3_ SC. The aim is to obtain a proper band alignment through the whole structure, not just at the carrier-transporting layer/absorber interface as usually encountered. This is performed by using a grading technique in the ETL, including linear, logarithmic, and parabolic BGG profiles along with doping grading within the absorber layer. The proposed structure is designed without a hole-transport layer, aiming to reduce interface and stability issues commonly associated with organic HTLs. Validation of the suggested model is needed to verify that the physical parameters are similar to the technological parameters. Accordingly, a calibration step is performed to validate the simulation reliability by benchmarking a simulated cell model against a similar fabricated cell whose structure is ITO/CdS/Sb_2_(S, Se)_3_/Au. Next, we replace the CdS ETL with Cd_1−x_Zn_x_S and vary the composition x of Zn to obtain graded ETL concentrations. These different compositions enable us to grade the bandgap from absorber to the FC and get the best alignment between them. Consequently, we choose the best absorber doping concentration (*N*_A_) and compare it with grading the acceptor doping concentration of the absorber. Finally, we optimize this cell by simultaneously selecting the optimal thickness (*t*_*abs*_) and the bulk defect density (*N*_*t*_). All this work is performed by the numerical simulator SCAPS-1D software under AM1.5G irradiation.

## Simulation method and main device structure

In this section, we introduce the HTL-free Sb_2_(S, Se)_3_ SC design, based on the configuration: ITO/CdS/Sb_2_(S, Se)_3_/Au. In doing our simulations, SCAPS-1D has been chosen as a device simulator platform^30^. Based on collected data from the literature, a validation step has been conducted through the calibration of simulated SC versus experiments. Some parameters are engineered to fit experimental data.

### SCAPS-1D

In this work, we used one dimensional numerical simulator SCAPS which has been progressed in Belguim (Gent university)^31^. Notably, SCAPS became a versatile and generally accepted tool that proved to be reliable, giving accurate simulation results which agree with the experimental investigations^31^. Poisson equation and continuity equations for hole and electron are the main equations used in SCAPS-1D. Auger and Shockley-Read-Hall (SRH) recombination models are implemented in it which are very essential for SC evaluation, especially in solar cells having high doping regions and high defect densities^18^. To get high performance and accurate simulation from it, other different factors also are considered like irradiance, temperature, and electrical bias conditions^32^. Current density–voltage (J-V), capacitance-voltage and -frequency (C-V, C-f) characteristics as well as External Quantum Efficiency (EQE) can be extracted from simulation helping for further optimization^32^.

### Device structure and its parameters

**Fig. 1:**
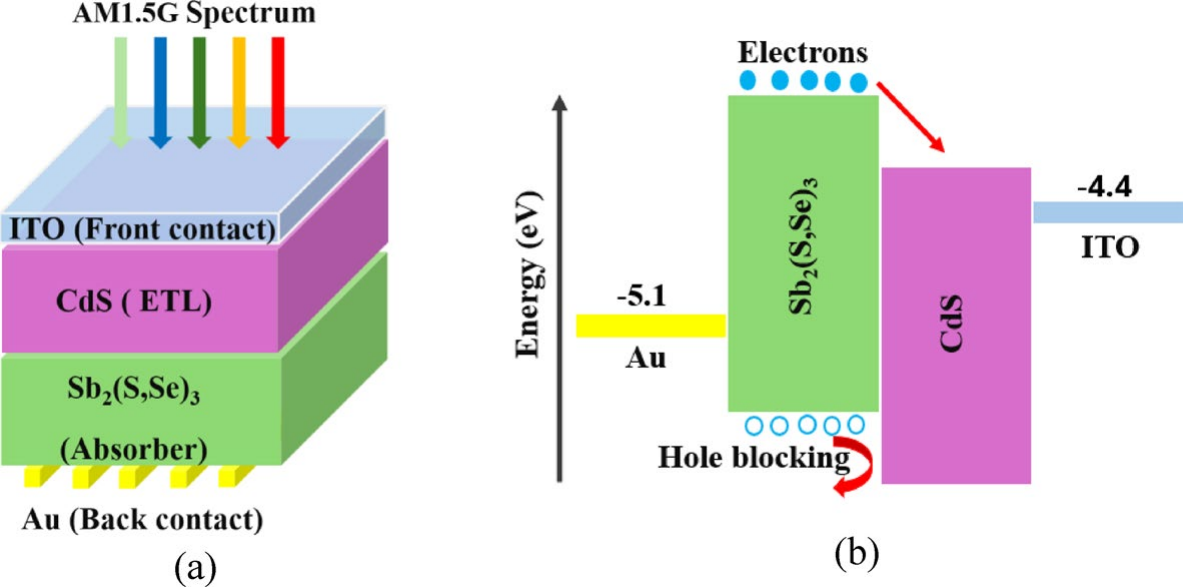
Sb_2_(S, Se)_3_ cell structure: (**a**) critical films ITO/CdS/Sb_2_(S, Se)_3_/Au, (**b**) CB and VB profile and flow of electrons and holes in cell.

The configuration and the energy bandgap diagram (EBD) of the device used are explained in Fig. 1. The solar cell contains a transparent front contact (ITO), ETL (CdS), absorber (Sb_2_(S, Se)_3_) and back electrode (Au), as shown in Fig. 1(a). The flow of free carriers (electrons and holes) according to the values of affinity and CBO from absorber to the ETL and rear contact is shown in Fig. 1(b). The conduction and valence band (CB &VB) are also shown. All the basic physical and technological factors of CdS and Sb_2_(S, Se)_3_ are illustrated in Table 1, which are acquired from previous experimental and simulation research^21,28^. The work function of the rear electrode is 5.1 eV, while that of the FC is 4.4 eV, as shown in Fig. 1(b). The introduced baseline HTL-free Sb_2_(S, Se)_3_ SC was fabricated according to X. Hu. and his team^33^. The CdS layer, serving as an ETL, was deposited on ITO utilizing chemical bath deposition (CBD). Then, VTD was employed to obtain Sb_2_(S, Se)_3_ thin film over CdS. The deposition of Au on the absorber film was finally done by thermal evaporation^33^.

An activation energy of recombination (*E*_*a*_) was measured to be 0.97 eV according to^16^. The electron affinity of absorber (χ_absorber_) is equal to 3.95 which fits the Sb_2_S_3_ mass ratio (ratio of S to S + Se) of 0.25^17^. Consequently, we can obtain a CBO value of −0.35 eV according to the relation^34^.

1 $$E_a =E_{g,abs} -\mid{\rm CBO} \mid$$

Where *E*_*g, abs*_ is the absorber bandgap. Also, knowing that^35^.

2 $${\rm CBO} = \Delta E_C = \chi_{\rm absorber}- \chi_{\rm ETL}$$

**Fig. 2:**
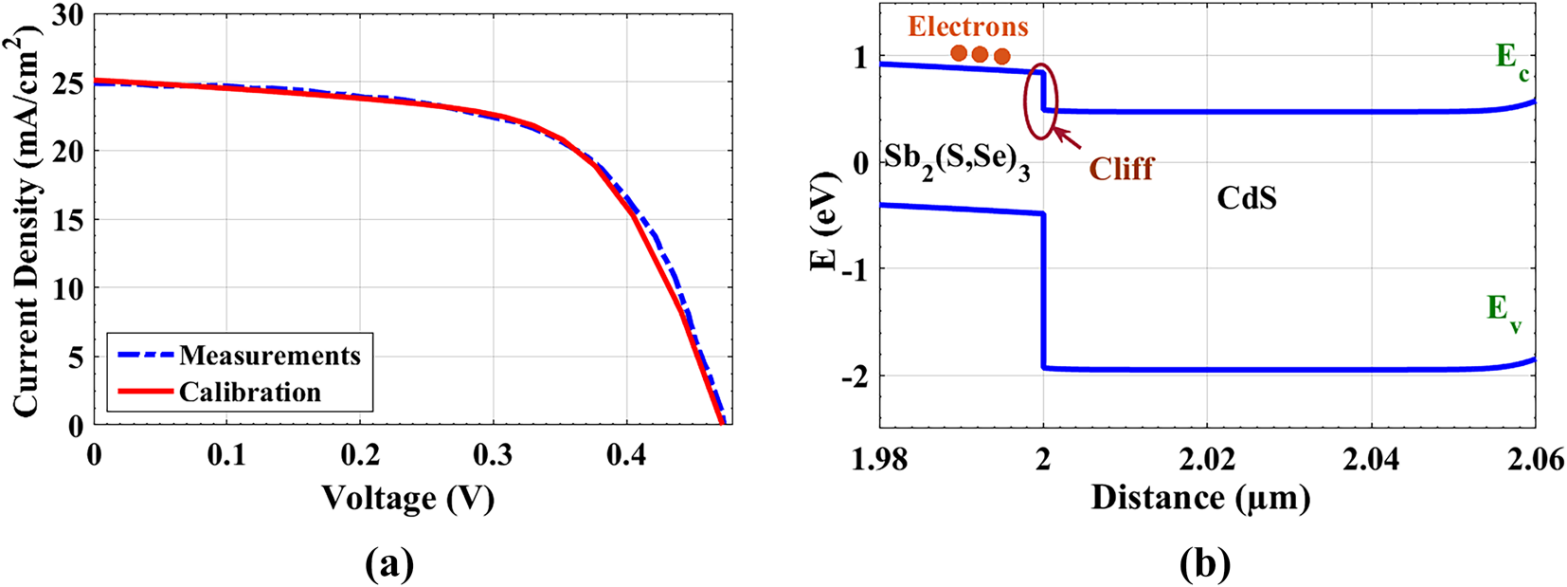
(**a**) Comparison of current density versus voltage curves for both experimental and calibrated Sb_2_(S, Se)_3_-based SCs without HTL, (**b**) Energy band profile of Sb_2_(S, Se)_3_ and CdS at illumination and at *J*_*sc*_ conditions.

Then, the electron affinity of CdS (χ_ETL_) is estimated to be 4.3 eV.

Table 2 shows Sb_2_(S, Se)_3_ bulk defect parameters with a high defect concentration of more than 1 × 10^14^ cm^−3^^28,36^. The absorption coefficients are extracted from literature^16^. Further, the defect factors of Sb_2_(S, Se)_3_/CdS interface are selected to give acceptable fitting against measurements with surface recombination velocity (SRV) of 1 × 10^7^ cm/s, as given in Table 3. Finally, the parasitic series resistance (*R*_*s*_) and the shallow uniform donor density *N*_D_ of CdS are supposed to be 2.3 Ω cm^2^ and 2.9 × 10^18^ cm^−3^, respectively, to fit the experimental *J-V* curve.

**Table 1:** Main parameters of the SC different layers^16,21,28,33,37,38^.

Material Parameters	CdS	CdZnS	Sb_2_(S, Se)_3_
Thickness (nm)	60	60	2000
Energy gap (eV)	2.42	2.42–3.54	1.325
Affinity (eV)	4.305	4.305–3.185	3.955
Relative permittivity	9	9	15
Hole/Electron mobility (cm^2^/V.s)	25/100	25/100	3/8
VB/CB effective DOS (cm^−3^)	1.8 × 10^19^/2.2 × 10^18^	1.8 × 10^19^/2.2 × 10^18^	1.8 × 10^18^/1 × 10^18^
Acceptor level (cm^−3^)	--	--	3.6 × 10^16^
Donor level (cm^−3^)	2.9 × 10^18^	2.9 × 10^18^	--

**Table 2:** Bulk defect factors for the Sb_2_(S, Se)_3_ layer^17,28,36,38^.

Defect Parameters	Defect 1	Defect 2	Defect 3
Type	Single acceptor (-/0)	Single acceptor (-/0)	Single donor (0/+)
Distribution	Uniform	Uniform	Uniform
Electron/hole capture cross sections (cm^2^)	1.0 × 10^−15^/ 1.5 × 10^−17^	1.0 × 10^−15^/ 4.9 × 10^−13^	4.0 × 10^−13^/ 1.0 × 10^−15^
Energy level (eV)	0.48 (above *E*_*v*_)	0.71 (above *E*_*v*_)	0.61 (below *E*_*c*_)
Density (*N*_*t*_) (cm^−3^)	1.2 × 10^15^	1.1 × 10^14^	2.6 × 10^14^

**Table 3:** Interfacial defect parameters ETL/Sb_2_(S, Se)_3_ layers^18^.

Interface Defect Parameters	ETL/Sb_2_(S, Se)_3_
Type	Neutral
Electron/hole capture cross sections (cm^2^)	1.0 × 10^−15^/1.0 × 10^−15^
Distribution	Single
Trap level with respect to highest *E*_*V*_ (eV)	0.65
Density (cm^−2^)	1 × 10^15^

**Fig. 3:**
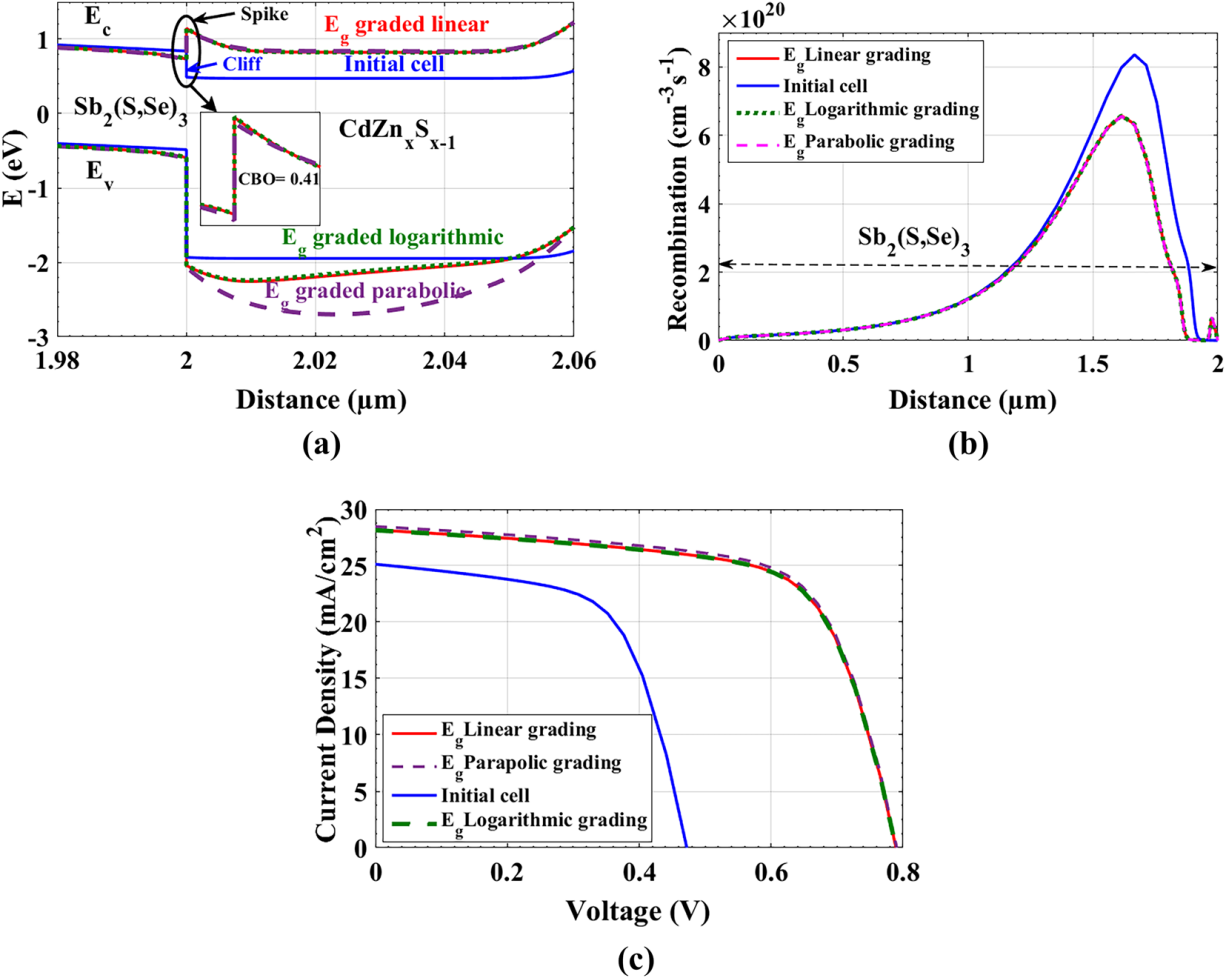
Comparison between BGG of Cd_1-x_ Zn_x_S (linear, logarithmic and parabolic) in illuminated HTL-free Sb_2_(S, Se)_3_ cells: (**a**) Energy bandgap bending in ETL and absorber illustrating the CBO on interface, (**b**) The recombination rate throughout the absorber, (**c**) *J-V* curves.

Based on the parameters mentioned above, the simulated *J-V* curve is shown in Fig. 3(a), in comparison to the experimental curve^17^. The open-circuit voltage (*V*_*oc*_), short-circuit current density (*J*_*sc*_), fill factor (FF), and PCE recorded from simulation and experimental work^33^ are listed in Table 4. This comparison indicates a decent agreement between experimental and simulated work. Alternatively, Fig. 3(b) presents the EBD, which is employed at short circuit and illumination conditions. *E*_*c*_ and *E*_*v*_ stand for the CB and VB edges, respectively. The EBD also shows how electrons move through the cliff from the absorber to the ETL. As can be seen from the EBD, the possibility of interfacial recombination at the ETL/absorber interface is high due to the low value of *E*_*a*_ (0.97 eV < *E*_*g, abs*_). This suggests that a large portion of charge carriers may be lost due to non-radiative recombination processes. To mitigate these recombination losses and enhance charge extraction, careful band alignment engineering is essential to improve electron transfer efficiency.

**Table 4:** Comparison between measurements and calibration factors of the Sb_2_(S, Se)_3_ cell without HTL.

PV parameters	V_oc_ (V)	J_sc_ (mA/cm^2^)	FF (%)	PCE (%)
Measurements	0.475	25.05	61.40	7.31
Calibration	0.474	25.12	61.41	7.31

## Result and discussion

The main purpose of employing suitable ETL and HTL is to accelerate the transition of electrons and holes from the absorber to the FC and back contact (BC), respectively, while inhibiting the movement of electrons and holes to the BC and FC as much as possible, respectively. Unfortunately, this produces electron and hole recombination at the ETL/absorber and HTL/absorber interfaces which lowers the SC performance. So, we utilize HTL-free design to minimize interface defects^27^. In addition, we propose a grading technique in ETL to accomplish an identifiable band alignment between ETL and absorber in order to facilitate the stream of electrons from absorber to the FC. We choose Cd_1−x_Zn_x_S as ETL material which enables us to get different affinities and an appropriate CBO. Different grading profiles (linear, parabolic, and logarithmic) are investigated. To obtain more optimized SC, we study the effect of changing other technological parameters of the absorber. One of the most effective parameters is the acceptor doping. We examine the most suitable doping concentration and compare it with doping grading technique to get the most effective electric field in the absorber. In addition, we investigate the impact of varying thickness utilizing various percentages of the original bulk defects on the achieved PCE.

### Bandgap grading profiles of Cd_1−x_Zn_x_S

Notably, the ternary compound Cd_1−x_Zn_x_S is a tunable bandgap material. It can be adjusted by varying the value of *x* to get the needed *E*_*g*_. This feature makes Cd_1−x_Zn_x_S a promising candidate for the use as an ETL, replacing the single bandgap CdS. Its tunable bandgap enables appropriate band alignment throughout the cell from the front contact to the absorber. Figure S1 represents the variation of χ and *E*_*g*_ of Cd_1−x_Zn_x_S w.r.t. the composition x (see Supporting file).

**Fig. 4:**
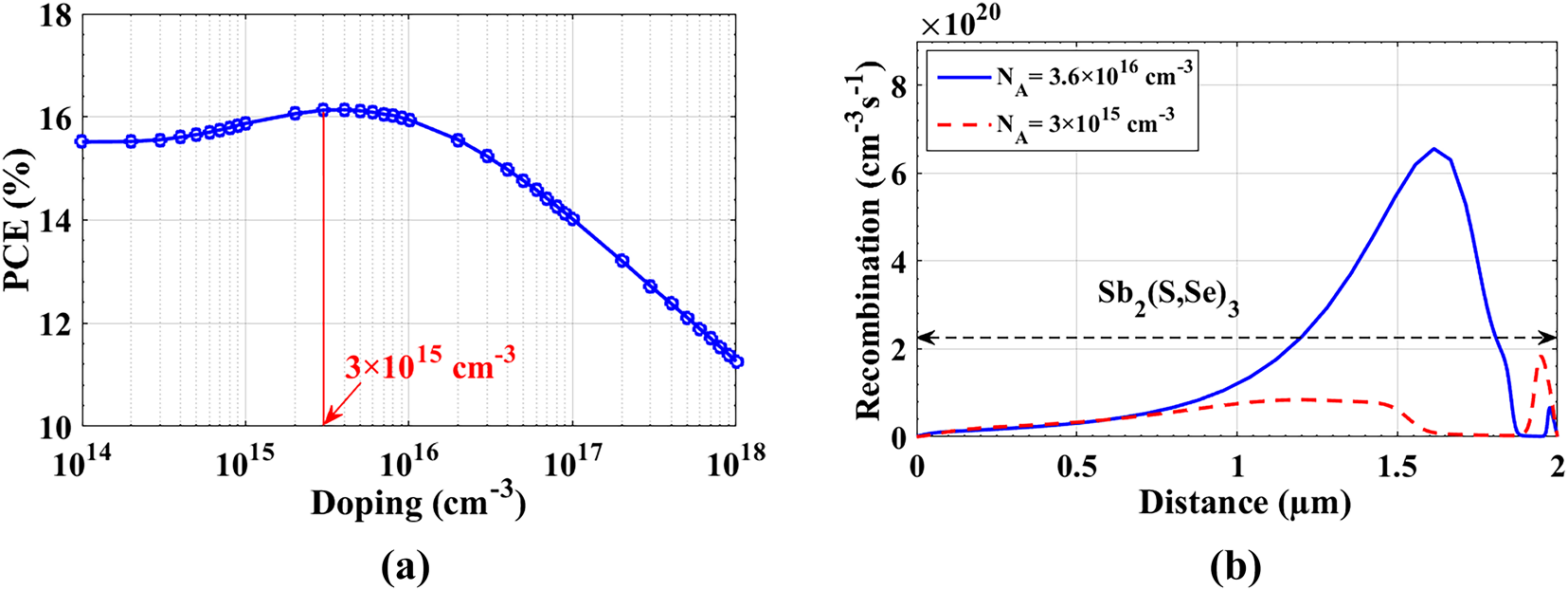
(**a**) Change of PCE of HTL-free Sb_2_(S, Se)_3_ cells with absorber doping concentration, and (**b**) Comparison between recombination rates at zero bias across the absorber cell with *N*_*A*_ = 3.6 × 10^16^ cm^−3^ and optimized doping cell with *N*_*A*_ = 3 × 10^15^ cm^−3^.

To start, we began from x = 0.5 at the right side to x = 0.81 at the left side. This permits us to get the preferable CBO between ITO/ETL and ETL/Absorber. Beginning from ITO to 3.955 eV affinity of absorber passing through BGG of ETL with two different affinities at the two right and left interfaces is applied on the cell. This grading helps us to use different ETL electron affinities and bandgaps in the two sides (right side/FTO and left side/absorber) of material. We used BGG technique with different profiles (linear, logarithmic, and parabolic), which is simulated using SCAPS software, as shown in Figure S2 (see Supporting file). The three profiles are almost giving the same behavior in the CB, but it differs a lot in the VB, especially for parabolic profile as shown in Fig. 4(a). This technique presents a positive spike of 0.41 eV between ETL/absorber with three different profiles compared to the cliff in the initial calibrated cell. The cliff (CBO < 0) does not impede the carrier transport, yet the interface recombination is the main mechanism in the cell. On the other side, the spike happens if CBO *>* 0 and acts as a barrier for photo-generated electrons. However, the spike of 0.41 eV is low enough to permit electrons in CB to flow to front contact and holes in VB to flow to the BC which lowers the photo-generated carrier recombination at interfaces. This makes the SRH recombination is the major mechanism in this structure. Consequently, this excellent flow of carriers leads to a decrease in bulk recombination at absorber as shown in Fig. 4(b). Lowering bulk recombination appears strongly in an increase in *J*_*sc*_. In addition, the mitigation of interface recombination leads to increasing *V*_*oc*_, as displayed in Fig. 4(c). The optimized cell with parabolic BGG of ETL acquires the best profile behavior with the following metrics: *V*_*oc*_ = 0.792 V, *J*_*sc*_ = 28.57 mA/cm^2^, FF = 66.84% and PCE = 15.07%, as illustrated in Table 5.

Figure 4. Comparison between BGG of Cd_1-x_ Zn_x_S (linear, logarithmic and parabolic) in illuminated HTL-free Sb_2_(S, Se)_3_ cells: (a) Energy bandgap bending in ETL and absorber illustrating the CBO on interface, (b) The recombination rate throughout the absorber, (c) *J-V* curves.

**Table 5:** Illuminated parameters between BGG of CdZn_x_S_1-x_ (linear, logarithmic and parabolic) of HTL-free Sb_2_(S, Se)_3_ structures.

PV parameters	V_oc_ (V)	J_sc_ (mA/cm^2^)	FF (%)	PCE (%)
Linear BGG	0.7909	28.159	66.74	14.86
Logarithmic BGG	0.7908	28.134	66.73	14.85
Parabolic BGG	0.7921	28.567	66.84	15.07

### Influence of the absorber doping concentration

#### Constant doping level optimization

**Fig. 5:**
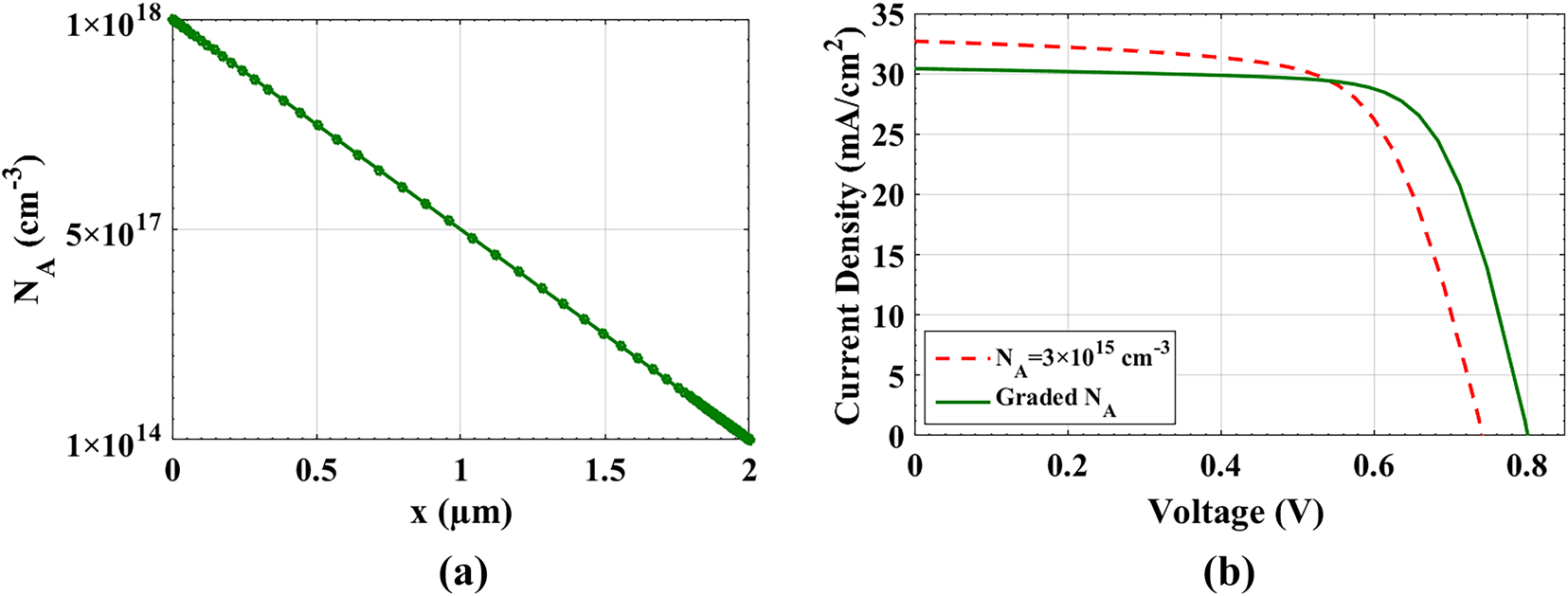
(**a**) Grading doping concentration (*N*_*A*_) along the absorber distance, and (**b**) comparison of J-V curves between optimized doping with *N*_*A*_ *=* 3 × 10^15^ cm^−3^ and cell with graded *N*_*A*_.

Here, the acceptor doping level of the absorber was made to change from *N*_*A*_ = 1 × 10^14^ cm^−3^ to 1 × 10^18^ cm^−3^ searching for enhancement of the performance of the cell. To obtain accurate results from this optimization, we used variable electron and hole mobility that is changing with every doping change as shown in Figure S3 (see Supporting file) following reference^13^. Figure 5(a) illustrates the change of the PCE versus absorber doping concentration. The optimum doping level is found to be *N*_A_ = 3 × 10^15^ cm^−3^, which gives the following PV parameters: *V*_*oc*_ = 0.743 V, *J*_*sc*_ = 32.69 mA/cm^2^, FF = 66.45% and PCE = 16.13%. The increase of PCE and *J*_*sc*_ is due to the effect of changing *N*_A_ to 3 × 10^15^ cm^−3^, which increases the carrier density of the absorber layer. However, excessively high doping concentrations can result in increased recombination rates due to carrier trapping effects as well as reduced carrier mobility. According to the easy photo-carrier transport from absorber, the bulk recombination is reduced, as established in Fig. 5(b).

#### Doping grading

**Fig. 6:**
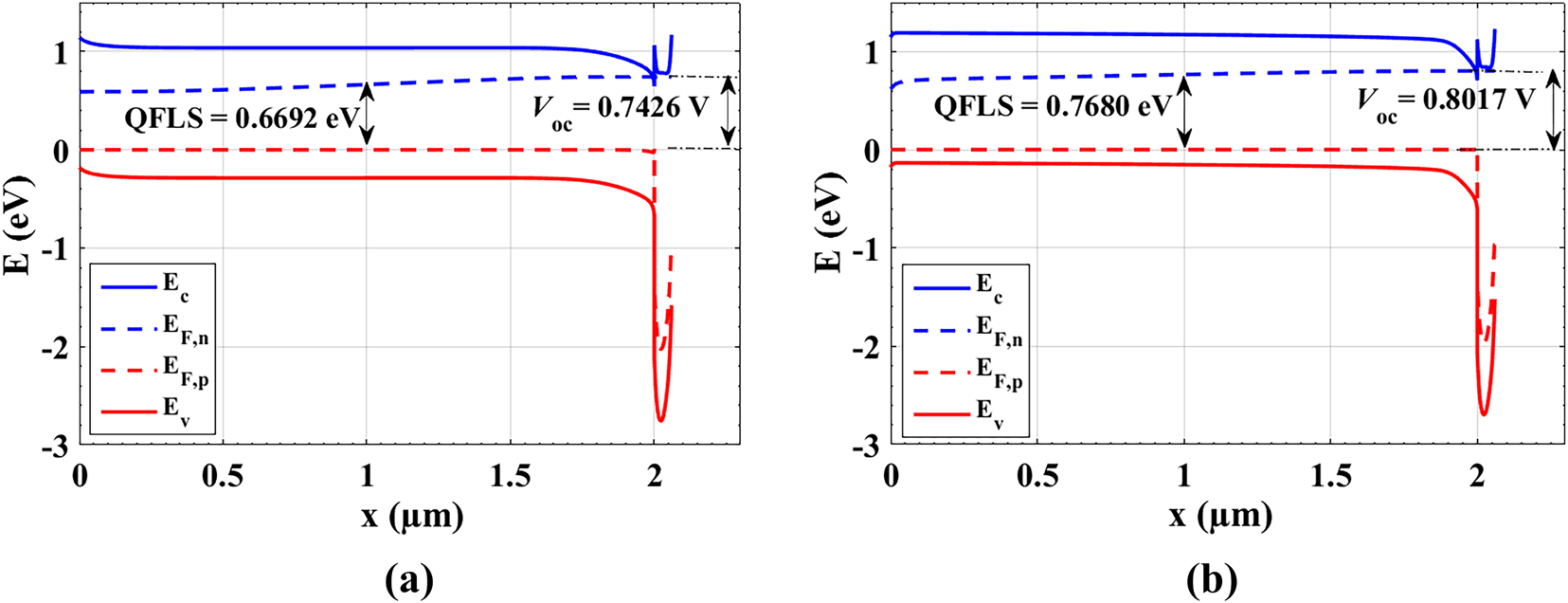
Energy band diagram and Quasi Fermi level gradient at open circuit at absorber with (**a**) *N*_A_ = 3 × 10^15^ cm^−3^ and (**b**) graded *N*_A_.

Here, we applied linear grading of doping starting with *N*_A_ = 1 × 10^14^ cm^−3^ at the right side to 1 × 10^18^ cm^−3^ at the left side of absorber as shown in Fig. 6(a). Increasing doping at the BC acts as a back surface field that can improve the extraction of carriers thereby boost the *V*_*oc*_. Figure 6(b) exhibits the J-V curves for two doping cases, namely, the linear grading doping technique and the fixed *N*_A_ = 3 × 10^15^ cm^−3^ case. While the grading technique results in lowering the value of *J*_*sc*_, other factors, FF and *V*_*oc*_, are enhanced, resulting in a rise in the PCE. The reduction of the *J*_*sc*_ is mainly due to the reduced mobility resulting from higher doping levels.

**Fig. 7:**
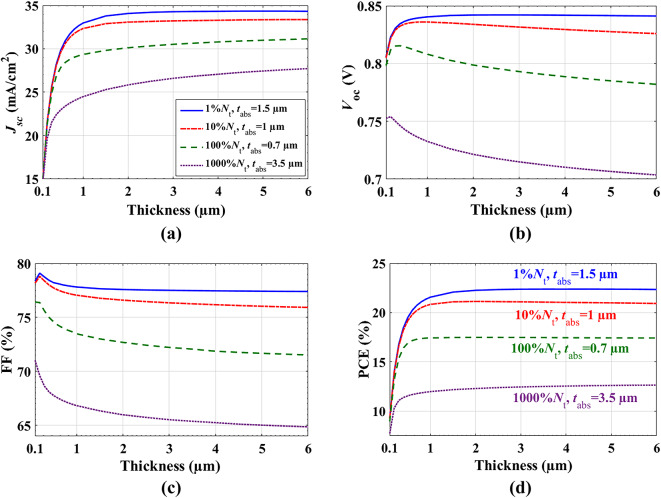
PV parameters variation at different thicknesses and bulk defect densities of absorber (**a**) *J*_*sc*_, (**b**) *V*_*oc*_, (**c**) FF, and (**d**) PCE.

The quantitative results, represented in Table 6, signify this conclusion. Figure 7 illustrates the energy band diagram (EBD) and Quasi Fermi level splitting (QFLS) at open-circuit conditions for two different absorber doping scenarios: (a) a uniformly doped absorber with *N*_*A*_ = 3 × 10^15^ cm^−3^ and (b) a graded doping profile for *N*_*A*_. In (a), the QFLS is 0.6692 eV, while in (b), it increases to 0.7680 eV, demonstrating that the graded doping enhances the separation of quasi-Fermi levels. Typically, a higher QFLS correlates with lower recombination rates and improved photovoltaic efficiency. This is clearly reflected in *V*_*oc*_, where the simulated *V*_*oc*_ values are 0.7426 V for the uniform doping case (a) and 0.8017 V for the graded doping case (b). The increase in *V*_*oc*_ for the graded absorber suggests better carrier collection and lower non-radiative recombination, leading to an overall enhancement in device performance.

**Table 6:** Illuminated parameters comparison between *N*_A_ = 3 × 10^15^ cm^−3^ and *N*_A_ grading.

PV parameters	V_oc_ (V)	J_sc_ (mA/cm^2^)	FF (%)	PCE (%)
*N*_A_=3 × 10^15^ cm^−3^	0.7426	32.692	66.45	16.13
Graded *N*_A_	0.8017	30.420	72.36	17.65

### Impact of absorber thickness and bulk defects

One of the key constraints that determines the PCE of a SC is the thickness of the absorber and the bulk trap density (*N*_t_). To avoid more carrier recombination, it is preferable to use a thickness that is shorter than the carrier’s diffusion length. However, higher absorption of sunlight necessitates a sufficiently large thickness. In addition, electron and hole recombination depends significantly on *N*_t_. Thus, examining the effect of absorber thickness in relation to total bulk traps is essential to achieving high PCE.

**Fig. 8:**
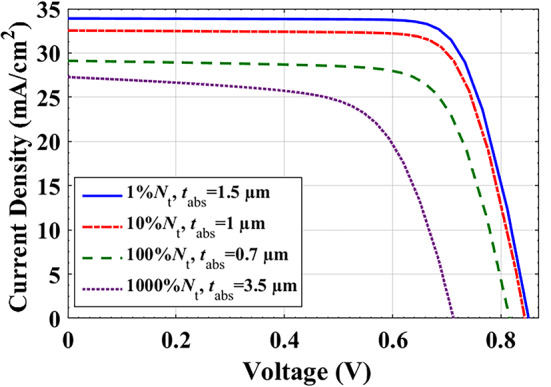
J*-V* curves for different thicknesses and bulk defects of absorber.

**Fig. 9:**
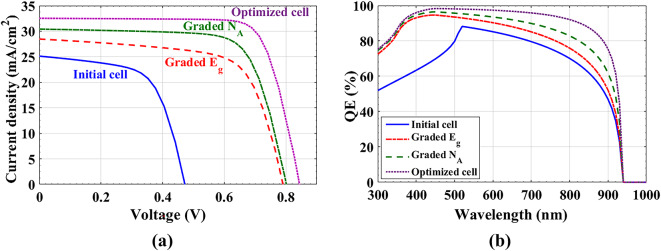
J-V for different optimization cases of the HTL free SC: (**a**) J-V curves. (**b**) quantum efficiency.

In these simulations, the absorber thickness is adjusted to be from 0.1 to 6 μm. Figure 8 shows the PV behavior of the cell at different bulk density percentages 1%, 10%, 100%, 1000% of the value used in calibration, *N*_t_. The contour plots for these changes are shown in Figure S4. For each value of *N*_*t*_, there is a certain absorber thickness at which the cell shows maximum efficiency. Figure 8(d) presents the best suggested *t*_*abs*_ (µm) and *N*_t_ (cm^−3^) for each case. Based on these choices, we plotted the J-V characteristics of four cells, as demonstrated in Fig. 9. The corresponding PV parameters are listed in Table 7. As seen, there is no extreme change in the PV parameters between cell 1 and cell 2. Therefore, the optimum PCE can be selected for cell 2 with the optimized parameters (10% *N*_t_, *t*_*abs*_ = 1 μm), which supports low-cost SC with acceptable total bulk defects and small thickness. The optimized metrics of cell 2 are: *V*_*oc*_ = 0.85 V, *J*_*sc*_ = 32.54 mA/cm^2^, FF = 76.88%, and PCE = 21.15%.

**Table 7:** PV factors of the simulated cell for different cases of thickness and bulk defects.

PV parameters	V_oc_ (V)	J_sc_ (mA/cm^2^)	FF (%)	PCE (%)
Cell 1 (1% *N*_*t*_, *t*_*abs*_ =1.5 μm)	0.85	33.90	77.46	22.41
Cell 2 (10% *N*_*t*_, *t*_*abs*_ =1 μm)	0.85	32.54	76.88	21.15
Cell 3 (100% *N*_*t*_, *t*_*abs*_ =0.7 μm)	0.82	29.12	73.63	17.52
Cell 4 (1000% *N*_*t*_, *t*_*abs*_ =3.5 μm)	0.71	27.27	65.23	12.69

### Final optimization

**Fig. 10:**
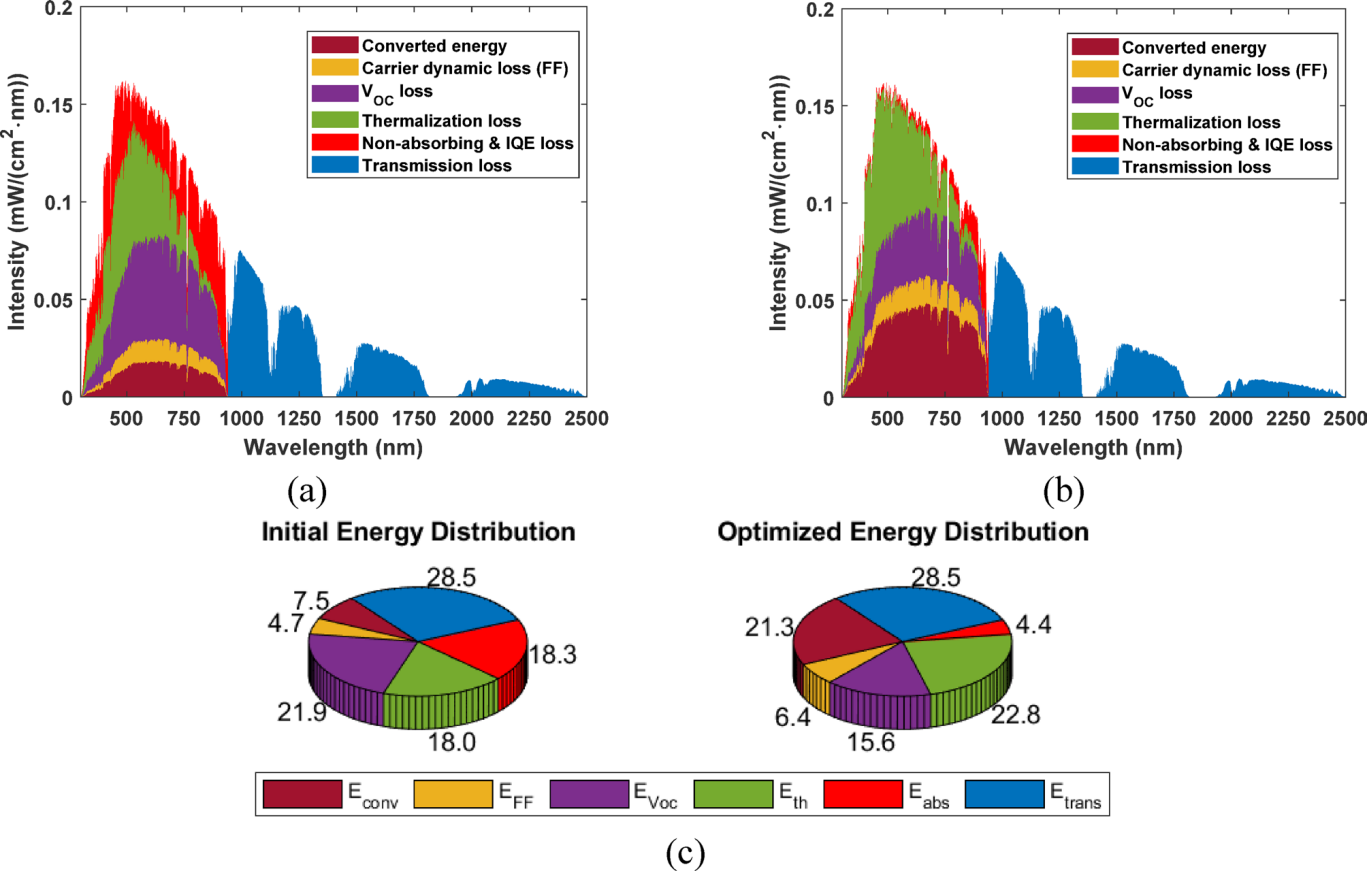
Spectral power density distribution shows the energy loss components in (**a**) the initial cell structure and (**b**) the optimized cell structure, while the integrated power loss is given for the two structures in (**c**).

All the above are trails to get more efficient solar cells with more photo-absorption and less bulk and interface recombination. The resulted optimized cell has absorber with physical parameters: *t*_*abs*_ = 1 μm, bulk defects has been decreased to 10% *N*_t_ cm^−3^, linear doping grading, and parabolic BGG of Cd_1−x_Zn_x_S as ETL. Figure 10(a) illustrates the *J-V* curves, and the improvement attained for *J*_*sc*_ and *V*_*oc*_ during the last steps with comparison to the initial cell. Table 8 presents a PV parameters’ comparison between all the previous optimized steps. Figure 10(b) shows the simulated external quantum efficiency (EQE) versus wavelength. As shown, the sunlight absorption of the initial cell is very weak in the 300–500 nm range. The optimized steps achieve significant progress in absorption, especially in the UV range.

**Table 8:** PV parameters’ comparison between all the previous optimized steps.

PV parameters	V_oc_ (V)	J_sc_ (mA/cm^2^)	FF (%)	PCE (%)
Measurements	0.475	25.05	61.4	7.31
Initial cell	0.474	25.12	61.41	7.31
Grad (ETL) E_g_ parabolic	0.792	28.56	66.84	15.07
Grad (Absorber) doping	0.801	30.42	72.36	17.65
Thickness & defects optimization	0.845	32.54	76.88	21.15

### Energy loss analysis

To assess energy losses in the analyzed cells, we separate the different energy losses following references^39,40^. The energy losses can be divided into (where $\:\varPhi\:\left(h\upsilon\:\right)$ is the solar energy spectrum and $\:h\upsilon\:$ is the photon energy):

- Transmission loss: $$\:{E}_{\text{t}\text{r}\text{a}\text{n}\text{s}}={\int\:}_{0}^{{E}_{\text{g}}}\varPhi\:\left(h\upsilon\:\right)\text{d}h\upsilon\:$$
- Insufficient light-absorbing: $\:{E}_{\text{a}\text{b}\text{s}}+{E}_{\text{I}\text{Q}\text{E}}={\int\:}_{{E}_{\text{g}}}^{4.43}\left(1-\text{E}\text{Q}\text{E}\left(h\upsilon\:\right)\right)\varPhi\:\left(h\upsilon\:\right)\text{d}h\upsilon\:$
- Thermalization energy loss: $\:{E}_{\text{t}\text{h}}={\int\:}_{{E}_{\text{g}}}^{4.43}\left(1-\frac{{E}_{\text{g}}}{h\upsilon\:}\right)\:\text{E}\text{Q}\text{E}\left(h\upsilon\:\right)\:\varPhi\:\left(h\upsilon\:\right)\:\text{d}h\upsilon\:$
- Open circuit voltage (*V*_*oc*_) loss: $\:{E}_{{V}_{\text{O}\text{C}}}={\int\:}_{{E}_{\text{g}}}^{4.43}\left[\frac{\left({E}_{\text{g}}-{V}_{\text{O}\text{C}}\right)}{h\upsilon\:}\right]\:\text{E}\text{Q}\text{E}\left(h\upsilon\:\right)\:\varPhi\:\left(h\upsilon\:\right)\:\text{d}h\upsilon\:$
- Filling Factor (FF) loss: $\:{E}_{\text{F}\text{F}}=\left(1-\text{F}\text{F}\right)\left({\int\:}_{{E}_{\text{g}}}^{4.43}\varPhi\:\left(h\upsilon\:\right)\text{d}h\upsilon\:-{E}_{\text{t}\text{r}\text{a}\text{n}\text{s}}-{E}_{\text{a}\text{b}\text{s}}-{E}_{\text{I}\text{Q}\text{E}}-{E}_{\text{t}\text{h}\text{e}\text{r}\text{m}}-{E}_{{V}_{\text{O}\text{C}}}\right)$

Figure 10 shows the energy loss components, while Table 9 compares all the distributed energy components of both initial and optimized SCs. In the optimized case, the absorption energy loss (*E*_abs_) has been significantly reduced from 18.316 W/cm^2^ (initial value) to 4.358 W/cm^2^. Notably, optimizing the absorber thickness and doping concentration reduces absorption loss by increasing the light absorption in the Sb_2_(S, Se)_3_ layer. On the other hand, there is an increase in thermal energy loss (*E*_th_) in the optimized structure, rising from 18.032 to 22.84 W/cm^2^. However, since the number of photons absorbed can impact the thermalization loss, we define the thermalization loss per electron (*E*_therm_/*N*_electrons_) as a more reasonable comparing metric. The average thermalization loss per electron in initial and optimized cells is found to be 0.719, and 0.701 eV, respectively. Thus, although the total thermalization loss is increased in the optimized cell due to the increased light absorption (as evident from the EQE in Fig. 10(b)), the thermalization energy loss per electron is reduced by distributing the light intensity among electrons.

Regarding open-circuit voltage energy loss (*E*_VOC_), *E*_VOC_ has decreased from 21.858 to 15.565 W/cm^2^, which is attributed to the optimized doping profile and reduced recombination. The fill-factor energy loss *E*_FF_ has increased in the optimized configuration from 4.726 to 6.402 W/cm^2^. Overall, the converted energy is shown to have a significant increase in the optimized configuration, from 7.5206 to 21.287 W/cm^2^, indicating a significantly higher overall energy conversion efficiency.

**Table 9:** Comparison between the distributed energy components of initial and optimized cells.

W/cm^2^	E_trans_	E_abs_	E_th_	E_VOC_	E_FF_	E_conv_
Initial	28.526	18.316	18.032	21.858	4.726	7.521
Optimized	28.526	4.358	22.84	15.565	6.402	21.287

Finally, Table 10 shows a comparison between some HTL-free Sb_2_(S, Se)_3_ structures that have been studied experimentally and theoretically over the last few years. This comparison illustrates the gradual progress made in research towards achieving a highly efficient solar cell with low cost, safe and easy fabrication. There are a lot of HTL-free cells that use different ETLs. Starting with H. Deng and his team in 2018, they made significant experimental progress from 1.8 to 5.6% PCE by using Sb_2_(Se_0.68_S_0.32_)_3_ as an absorber and TiO_2_ as an ETL. Furthermore, Ishaq et al. 2018 experimentally introduced double buffer layer ZnO/CdS for the first time in the Sb_2_(Se_0.68_S_0.32_)_3_ system which improves efficiency from 4.17 to 5.73%. In 2021, Xiaobo Hu et al., fabricated Sb_2_(S_0.25,_ Se_0.75_)_3_ thin films using the VTD method and got 7.31% PCE. For the band alignment at the ETL/absorber interface, others used different ETLs to modify the CBO. M. S. Salem et al., in 2022, utilized Zn_0.85_Mg_0.15_O in HTL-free SC and achieved 20% PCE.

**Table 10:** A state-of-the-art quantitative comparison between Sb_2_(S, Se)_3_ cells without HTL performed by experimental and simulated studies.

Reference	ETL	Approach	PCE (%)
^29^	TiO_2_	Experimental	5.6%
^33^	CdS	Experimental	7.31
^9^	CdS	Experimental	6.30
^41^	ZnO/CdS	Experimental	5.73%
^18^	Cd_0.44_Zn_0_._56_S	Simulation	14.86%
^27^	Cd_0.44_Zn_0_._56_S	Simulation	21.86.
^2^	Zn_0.85_Mg_0.15_O	Simulation	20
This work	*CdS*	Simulation	7.31
This work	*E*_*g*_ Graded Cd_1−x_Zn_x_S	Simulation	15.07
This work	*E*_*g*_ Graded Cd_1−x_Zn_x_S, *N*_*A*_ = 3 × 10^15^ cm^−3^	Simulation	16.13
This work	*E*_*g*_ Graded Cd_1−x_Zn_x_S, Graded *N*_*A*_	Simulation	17.65
This work	*E*_*g*_ Graded Cd_1−x_Zn_x_S, Graded *N*_A_, 10% *N*_t_, *t*_*abs*_ =1 μm	Simulation	21.15

## Conclusions

In this paper, we introduced single junction SC based on Sb_2_(S, Se)_3_ as an absorber. The cell is designed as HTL-free for low cost and more stability. The applied cell is simulated by the numerical simulator SCAPS-1D. The Simulation parameters are adjusted to satisfy good agreement comparable to the experimental data. The simulated cell performance is investigated under various effects such as CBO adjustment between ETL and absorber, absorber doping concentration, absorber thickness and bulk trap defects. It was concluded that CBO is the most effective parameter. To get more effective CBO for this cell, we replaced the CdS with the tunable bandgap CdZnS composite. Optimizing CBO is applied by ETL parabolic grading technique. Choosing two different compositions of Zn (0.5, 0.81) at the two interfaces enables to grade the electron affinity from absorber to front contact. This ETL grading improved performance drastically (more than 100%) relative to the initially simulated cell. The optimized cell parameters satisfy *V*_oc_ = 0.792 V, *J*_sc_ = 28.56 mA/cm^2^, FF = 66.84% and PCE = 15.07%. Furthermore, the acceptor doping is optimized by grading it across the absorber from 1 × 10^15^ to 1 × 10^18^ cm^−3^. This step raises the PCE to 17.65% due to the increase in the difference between electron and hole fermi levels despite increasing the *J*_sc_. More ever, the influence of thickness and trap defects of the absorber are also investigated. Reducing the thickness to one half (*t*_*abs*_ =1 μm) with bulk defects of 10% of the initial defects result in the following PV parameters: *V*_oc_ = 0.845 V, *J*_sc_ = 32.54 mA/cm^2^, FF = 76.88%, and PCE = 21.15%.

This study promotes the usage of the HTL-free Sb_2_(S, Se)_3_ SC design and points to various techniques for the optimization of solar cell performance by grading numerous parameters to control the flow of electrons between the layers and to achieve higher efficiency along with predicted low-cost and higher stability.

## Electronic supplementary material

Below is the link to the electronic supplementary material.

Supplementary Material 1

## Data Availability

All data that support the findings of this study are included within the article (and any supplementary files).
