# Supplementary material for: Unveiling the role of dual grading in device optimization of HTL-free Sb2(S, Se)3 solar cells
Source: Sci Rep. 2025 Jul 25;15:27050. doi: 10.1038/s41598-025-11658-8 (PMC12297243; doi:10.1038/s41598-025-11658-8)
Supplement: Supplementary file 1 — Supplementary Material 1 [file 41598_2025_11658_MOESM1_ESM.pdf]

Supporting Information

HTL-free $Sb_2(S,Se)_3$ Solar Cells: Unveiling the Role of Dual Grading in Device Optimization

Basma A. A. Osman, Ahmed Shaker, Ibrahim S. Ahmed and Tarek M. Abdolkader

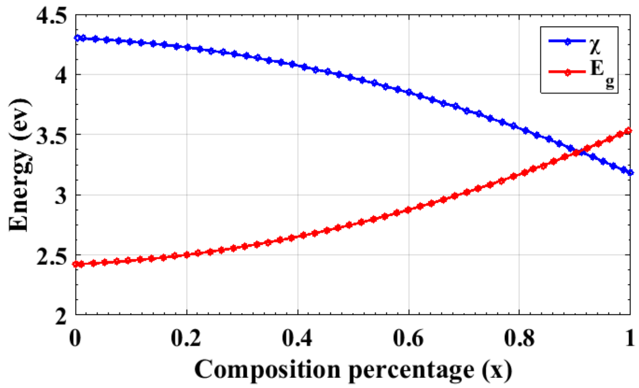

Figure S1 Variation of electron affinity and energy bandgap with the Zn composition of $Cd_{1-x}Zn_xS$.

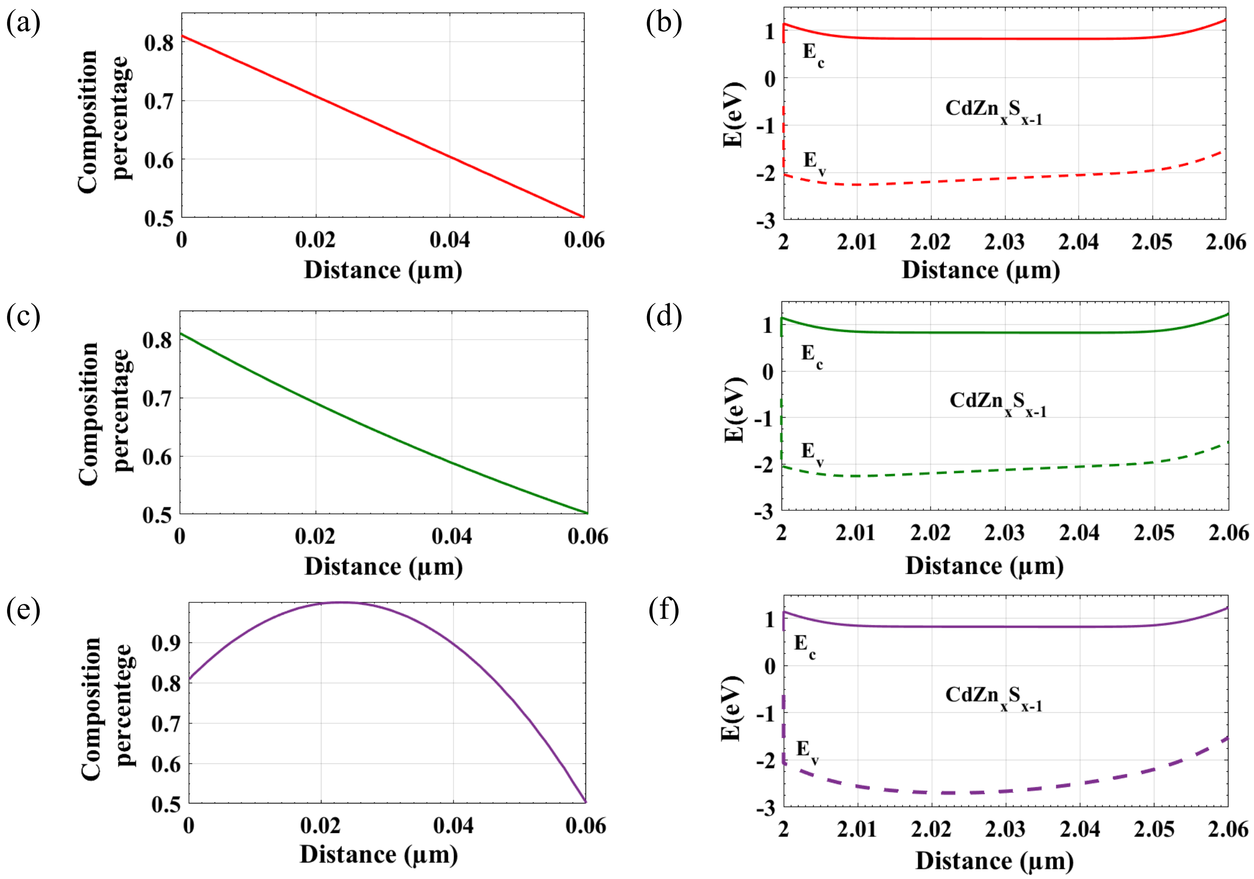

Figure S2 Different grading profiles of the Zn composition of $Cd_{1-x}Zn_xS$ with position in the layer: (a) linear, (c) logarithmic and (e) negative parabolic, The energy band gap diagram in the three grading profiles: (b) linear, (d)logarithmic and (f) negative parabolic.

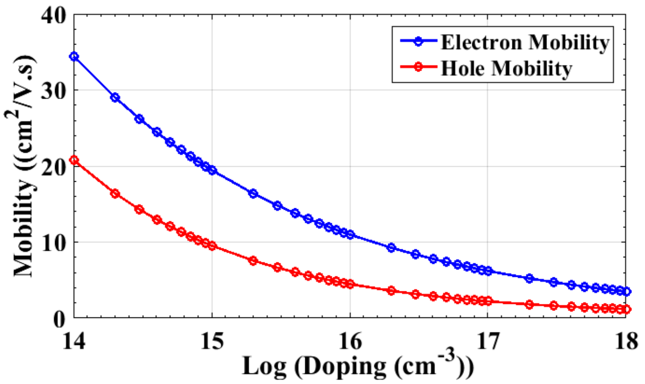

Figure S3 $Sb_2(S,Se)_3$ electron and hole mobility versus doping concentration.

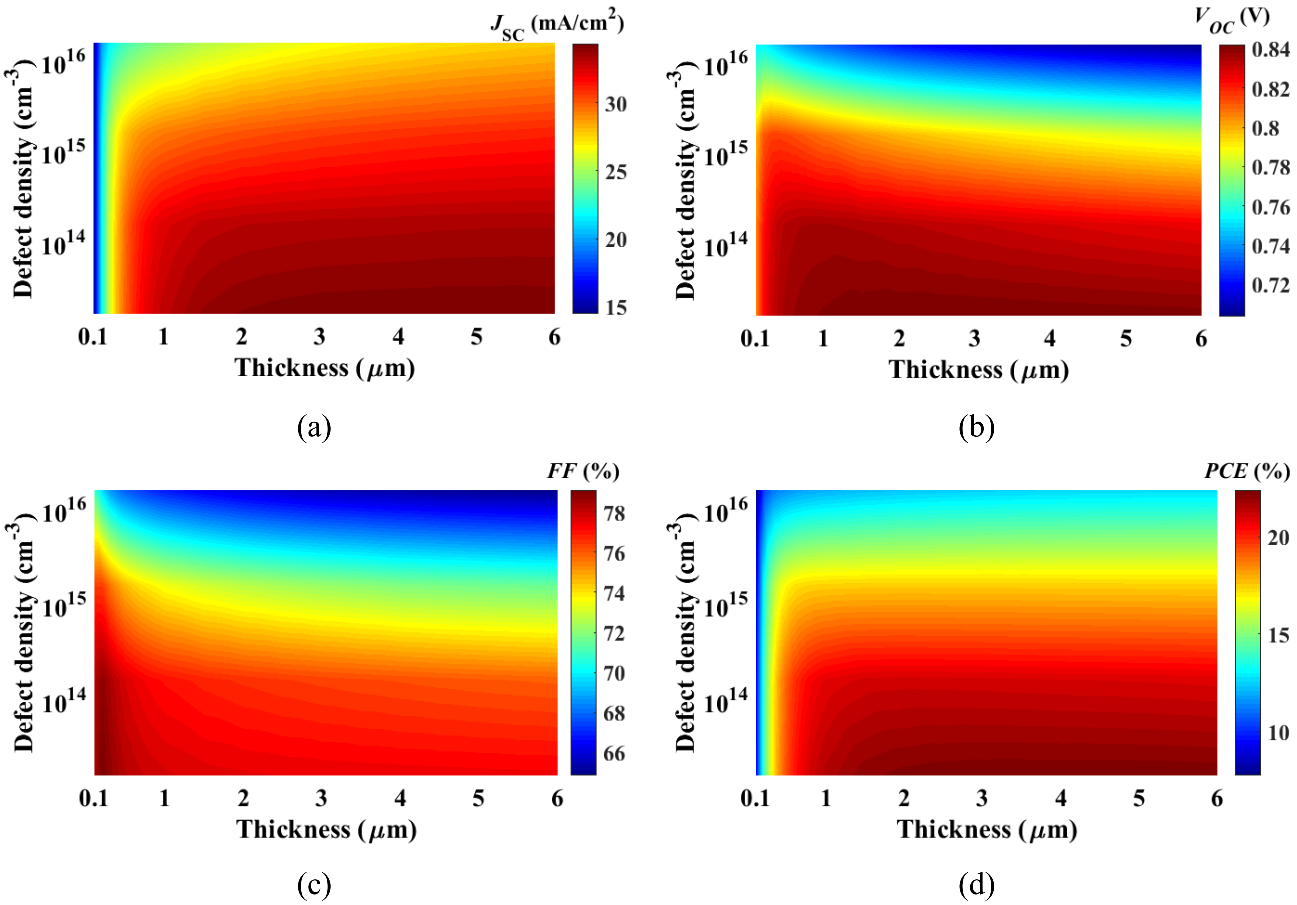

Figure S4 Contour of PV parameters variation at different thicknesses and bulk defect densities of absorber (a) J_{sc}, (b) V_{oc}, (c) FF, and (d) PCE.
